# Suitability of hepatocyte cell lines HepG2, AML12 and THLE-2 for investigation of insulin signalling and hepatokine gene expression

**DOI:** 10.1098/rsob.180147

**Published:** 2018-10-24

**Authors:** Stephanie Sefried, Hans-Ulrich Häring, Cora Weigert, Sabine S. Eckstein

**Affiliations:** 1 Division of Pathobiochemistry and Clinical Chemistry, Department of Internal Medicine IV, University Hospital Tübingen, Tübingen, Germany; 2 German Center for Diabetes Research (DZD), München-Neuherberg, Germany; 3 Institute for Diabetes Research and Metabolite Diseases of the Helmholtz Centre Munich at the University of Tübingen, Tübingen, Germany

**Keywords:** insulin, hepatocyte, signalling, cells

## Abstract

Immortal hepatocyte cell lines are widely used to elucidate insulin-dependent signalling pathways and regulation of hepatic metabolism, although the often tumorigenic origin might not represent the metabolic state of healthy hepatocytes. We aimed to investigate if murine cell line AML12 and human cell line THLE-2, which are derived from healthy liver cells, are comparable to hepatoma cell line HepG2 for studying acute insulin signalling and expression of gluconeogenic enzymes and hepatokines. Insulin responsiveness of AML12 and THLE-2 cells was impaired when cells were cultured in the recommended growth medium, but comparable with HepG2 cells by using insulin-deficient medium. THLE-2 cells showed low abundance of insulin receptor, while protein levels in HepG2 and AML12 were comparable. AML12 and THLE-2 cells showed only low or non-detectable transcript levels of *G6PC* and *PCK1*. Expression of *ANGPTL4* was regulated similarly in HepG2 and AML12 cells upon peroxisome proliferator-activated receptor δ activation but only HepG2 cells resemble the *in vivo* regulation of hepatic *ANGPTL4* by cAMP. Composition of the culture medium and protein expression levels of key signalling proteins should be considered when AML12 and THLE-2 are used to study insulin signalling. With regard to gluconeogenesis and hepatokine expression, HepG2 cells appear to be closer to the *in vivo* situation despite the tumorigenic origin.

## 1. Background

The liver plays a key role in energy homoeostasis by regulation of glucose and lipid metabolism and synthesis of hepatokines. The liver maintains a normal blood glucose level during fasting by breakdown of glycogen and by gluconeogenesis with subsequent release of glucose into the systemic circulation. Postprandial glucose is taken up into the liver and hepatic insulin signalling via the AKT pathway leads to various effects such as glycogen storage, inhibition of gluconeogenesis, promotion of lipogenesis and protein synthesis, and activation of pro-survival pathways. Hepatokines are liver-derived proteins that regulate metabolic pathways in other tissues and have attracted much interest in the last few years since they are in focus for the development of drugs targeting diabetes [1,2]. The comprehensive understanding of the signalling pathways that regulate the metabolic functions in the liver is highly important to target the disturbances in pathological states. Hepatic cell culture models are indispensable in this context. Undoubtedly primary cells are superior over permanent cell lines since they resemble more the *in vivo* situation. However, the availability of primary hepatocytes is limited, especially human primary hepatocytes are rarely accessible. In addition, the phenotype is unstable and primary cells can only be cultured for a short time span [3,4]. Permanent cell lines have several advantages such as immortality and the possibility to easily interfere with the abundance and activity of potential regulators of metabolic pathways. Cell lines originating from hepatic tumours are immortal but also cells from healthy organs can be artificially immortalized with a variety of methods. In general, liver cell lines are often used for studies on xenobiotic metabolism and hepatotoxicity, and the focus is drawn towards enzyme capacities [5]. In diabetes research, the signalling pathways that regulate hepatic glucose and lipid metabolism are of great interest. The human hepatoma cell line HepG2 is frequently used to investigate insulin-dependent pathways [6], but these cells are derived from a Caucasian male with a differentiated hepatocellular carcinoma [7] and the origin from tumour tissue influences the metabolic phenotype. Investigation of the HepG2 proteome revealed e.g. impairments in gluconeogenesis, fatty acid oxidation and greater reliance on non-oxidative glucose metabolism compared with primary human hepatocytes [8,9]. Hepatocyte cell lines derived from healthy liver tissue might be closer to primary cells, but the insulin responsiveness of many available hepatocyte cell lines is not characterized. Murine hepatocyte cell line AML12 is derived from liver of transgenic mice overexpressing transforming growth factor (TGF) α [10] and has mainly been used for studies on lipid metabolism extending to steatosis/non-alcoholic fatty liver disease [11–13] and liver injury [14–16]. THLE-2 cells were obtained from human adult hepatocytes and were immortalized by introduction of simian virus 40 large T antigen [17]. These cells are mainly used to study cytotoxic agents [18,19]. We characterized here the suitability of AML12 [10] and THLE-2 [17] cells to investigate aspects of insulin signalling and regulation of gluconeogenic enzymes and hepatokines, and compared them with HepG2 cells. We took into account that the growth media of the three cell lines differ markedly in their insulin content, and used also media with comparable insulin concentrations for the experiments.

## 2. Results

### 2.1. Phosphorylation of AKT after acute insulin stimulation

**Figure 1.**
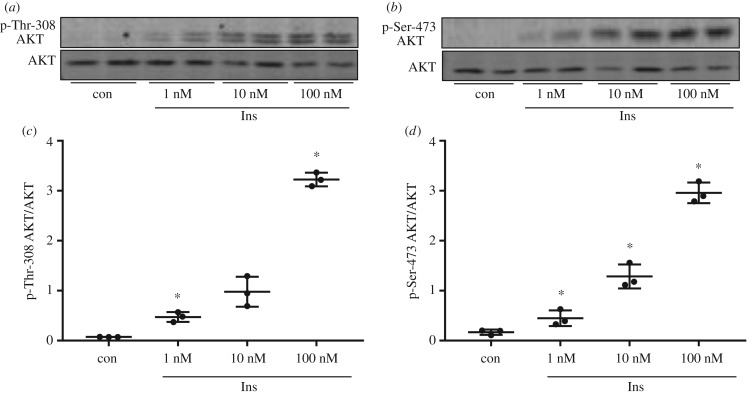
Insulin stimulation in HepG2 cells. (*a*,*b*) HepG2 cells were cultured without serum for 3 h before stimulation with 1, 10 and 100 nM insulin (Ins) for 10 min. 20 µg protein was analysed by western blot for the presence of p-Thr-308/p-Ser-473 of AKT and AKT protein. (*c*,*d*) Band intensities of phosphorylated AKT were normalized for AKT protein content (*n* = 3; mean ± s.d.; *p < 0.05, control (con) versus 1 nM, 1 versus 10 nM, 10 versus 100 nM).

Insulin responsiveness was studied as phosphorylation of Thr-308 and Ser-473 of AKT after acute insulin stimulation for 10 min. HepG2 cells showed significantly increased AKT phosphorylation at both sites after stimulation with 1 nM insulin for 10 min that was further increased with 10 and 100 nM insulin (figure 1).

**Figure 2.**
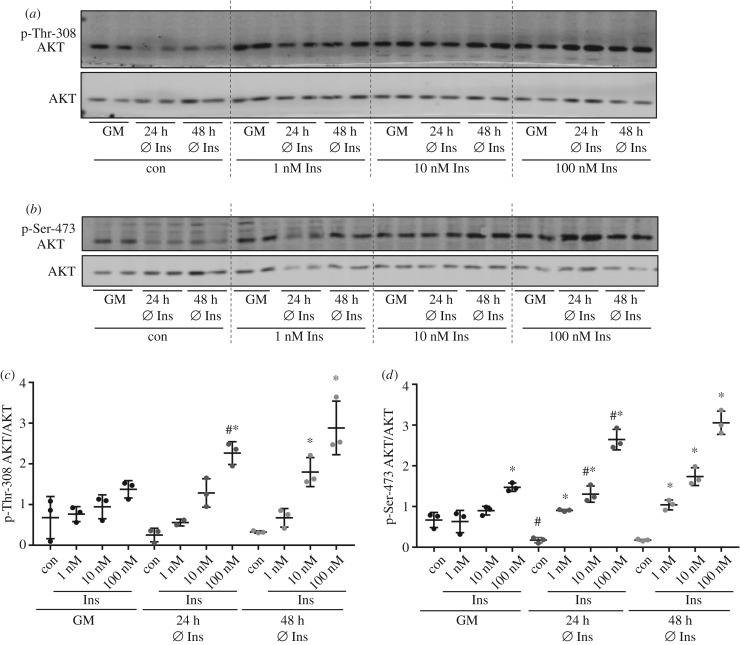
Insulin stimulation in AML12 cells. AML12 cells were either grown in normal growth medium (GM) with all supplements or cultured for 24 or 48 h without the supplementation of insulin in the culture medium. Cells were serum starved for 3 h before stimulation with 1, 10 or 100 nM insulin for 10 min. (*a*,*b*) Western blot analysis with 20 µg of total protein was performed to detect p-Thr-308/p-Ser-473 of AKT and AKT. (*c*,*d*) Band intensities of phosphorylated AKT were normalized for AKT protein content (*n* = 3; mean ± s.d.; *p < 0.05, con versus 1 nM, 1 versus 10 nM, 10 versus 100 nM; ^#^*p* < 0.05, GM versus 24 h Ø Ins con or at respective insulin concentration). Dashed lines separate samples with different insulin concentration.

When AML12 cells were stimulated with insulin in the recommended growth medium containing 850 nM insulin [10], only a marginal increase of phosphorylation was achieved reaching significance for Ser-473 after stimulation with 100 nM insulin (figure 2). Withdrawal of insulin from the growth medium for 24 h led to significant increase of AKT phosphorylation on both sites after acute insulin stimulation compared with insulin-stimulated cells that were cultured in normal growth medium (figure 2*c*,*d*). In addition, removal of insulin from the culture medium resulted in a reduced basal phosphorylation of AKT (figure 2*d*). Withdrawal of insulin for 48 h during cultivation did not further increase the response in AKT phosphorylation.

**Figure 3.**
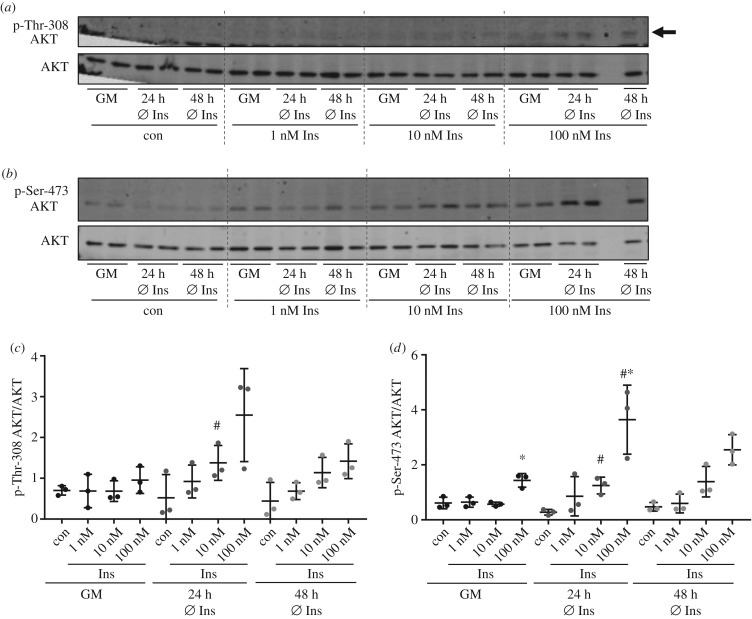
Insulin stimulation in THLE-2 cells. THLE-2 cells were either grown in normal growth medium (GM) with all necessary supplements or cultured for 24 or 48 h without the supplementation of insulin in the culture medium. All cells were serum starved for 3 h before stimulation with 1, 10 or 100 nM insulin for 10 min. (*a*,*b*) Western blot analysis with 20 µg of total protein was performed to detect p-Thr-308/p-Ser-473 of AKT and AKT. Arrow indicates p-Thr-308. No sample was loaded in the penultimate lane. (*c*,*d*) Band intensities of phosphorylated AKT were normalized for AKT protein content (*n* = 3; mean ± s.d.; *p < 0.05, 10 versus 100 nM; ^#^*p* < 0.05, GM versus 24 h Ø Ins at respective insulin concentration). Dashed lines separate samples with different insulin concentration.

Growth medium of THLE-2 cells also contains high insulin concentrations (800–850 nM insulin) as recommended by the manufacturer (www.lgcstandards-atcc.org) and, similarly to AML12 cells, only phosphorylation of Ser-473 of AKT was significantly increased using 100 nM insulin (figure 3). Withdrawal of insulin for 24 h increased the acute effect of insulin and phosphorylation was significantly higher for Thr-308 after stimulation with 10 nM insulin and for Ser-473 after stimulation with 10 and 100 nM insulin (figure 3*c*,*d*). In line with AML12 cells, cultivation without insulin for 48 h did not result in a further increase of AKT phosphorylation in THLE-2 cells.

**Figure 4.**
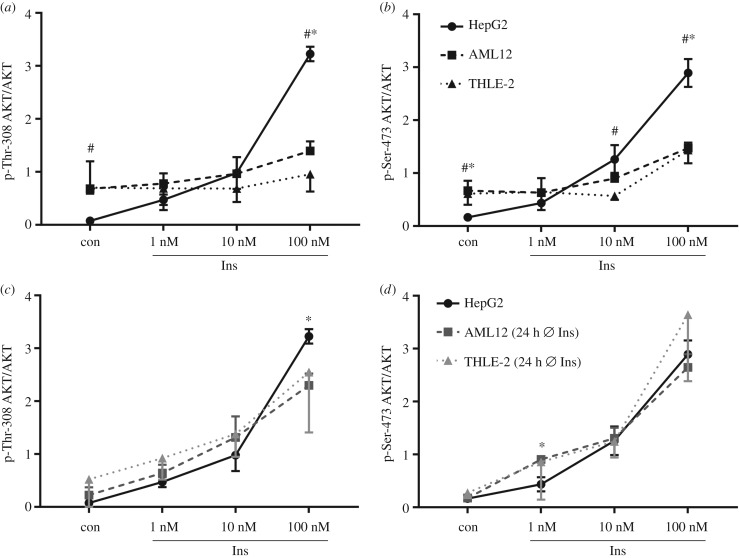
Comparison of AKT phosphorylation between the different cell lines. Signal intensities of phosphorylated AKT normalized for AKT protein amount as shown in figures 1–3 were compared between the different cell lines. Identical data of HepG2 cells were included in (*a*,*c*) and (*b*,*d*). (*a*,*b*) Cells were grown in their respective growth medium as recommended. (*c*,*d*) HepG2 cells were grown in their growth medium whereas AML12 and THLE-2 cells were cultured for 24 h without additional insulin (*n* = 3; mean ± s.d.; *p < 0.05, HepG2 versus AML12; ^#^*p* < 0.05, HepG2 versus THLE-2).

Figure 4 compares ratios of phosphorylated AKT to AKT between the different cell lines and cultivation conditions. When AML12 and THLE-2 cells were grown in their respective growth medium supplemented with insulin, basal AKT phosphorylation was higher when compared with basal levels of AKT phosphorylation in HepG2 cells, but the extent of insulin-induced phosphorylation of AKT was clearly reduced (figure 4*a*,*b*). Cultivation in insulin-deficient medium for 24 h reduced basal AKT phosphorylation and increased the extent of AKT phosphorylation on Thr-308 and Ser-473 in AML12 and THLE-2 cells after insulin stimulation to a level that is comparable to HepG2 cells (figure 4*c*,*d*). To conclude, when cultivated in media containing equally low insulin concentrations, AML12 and THLE-2 cells showed similar AKT phosphorylation in response to insulin compared with HepG2 cells.

### 2.2. Protein abundance of IRS1, IRS2 and IR

**Figure 5.**
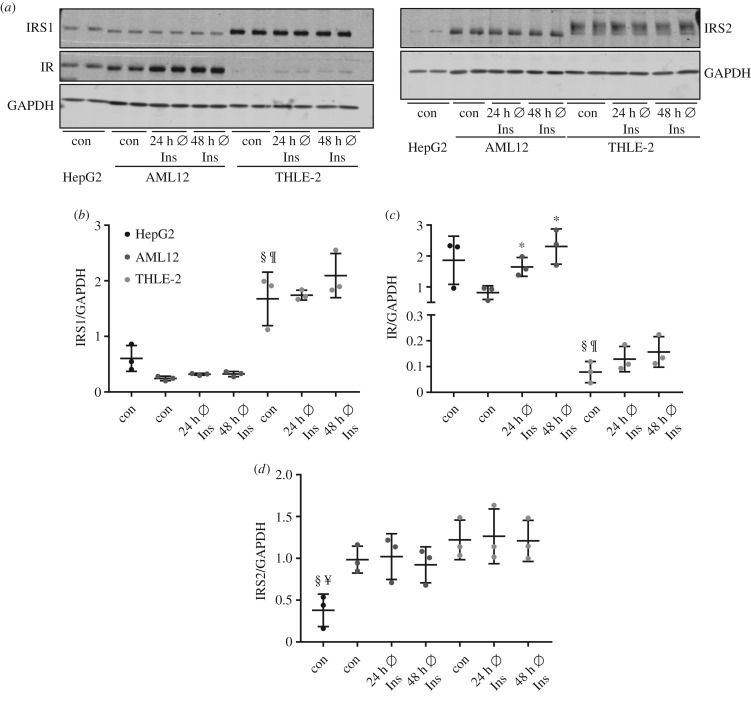
Protein abundance of IRS1, IRS2 and IR in HepG2, AML12 and THLE-2 cells. Cells were grown in their respective growth medium (con) or cultured for 24/48 h without additional insulin. (*a*) 15 µg protein was separated and checked by western blot for the presence of IRS1, IRS2 and IR. GAPDH was used as loading control. (*b*–*d*) Band intensities were normalized for GAPDH (*n* = 3; mean ± s.d.; *p < 0.05, con versus 24/48 h Ø Ins in AML12 cells; ^§^*p* < 0.05, con HepG2 versus con THLE-2; ^¶^*p* < 0.05, con AML12 versus con THLE-2; ^¥^*p* < 0.05, con HepG2 versus con AML12).

Protein abundance of key components of the insulin signalling pathway in the cell lines was investigated. THLE-2 cells showed the highest level of insulin receptor substrate (IRS)1 compared with HepG2 and AML12, but a significantly lower signal for the insulin receptor (IR) (figure 5*a*–*c*). Notably, withdrawal of insulin for 24 and 48 h significantly increased the level of IR protein in AML12 and a similar trend was observed in THLE-2 cells (figure 5*c*). Abundance of IRS2 is similar in AML12 and THLE-2 cells and significantly lower in HepG2 cells (figure 5*d*).

### 2.3. Expression of gluconeogenic genes

**Figure 6.**
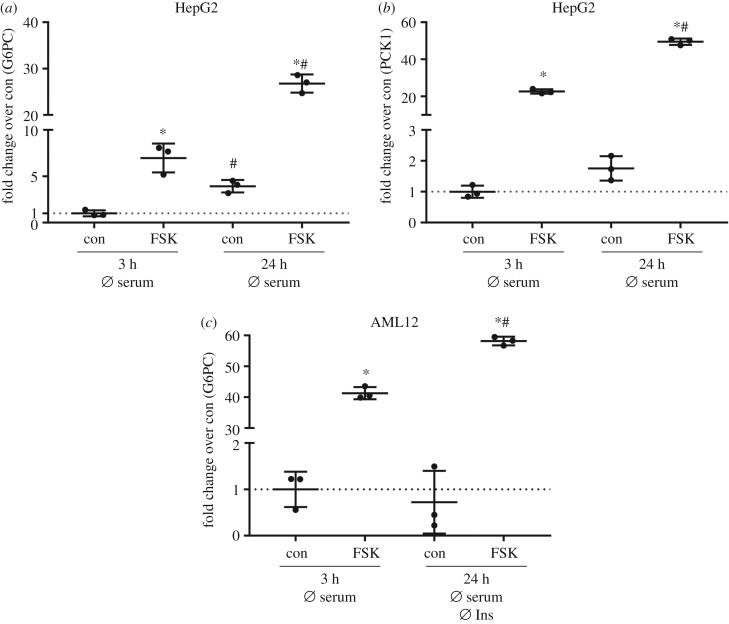
Expression of gluconeogenic genes in HepG2 and AML12 cells. (*a*,*b*) HepG2 cells were deprived of serum for 3 h or for 24 h before stimulation with 20 µM forskolin (FSK) for 2 h. RNA isolation and cDNA synthesis followed qPCR to measure mRNA abundance of *G6PC* (*a*) and *PCK1* (*b*). (*c*) AML12 cells were grown in normal growth medium and deprived of serum for 3 h before stimulation with 20 µM FSK for 2 h. A subset of cells was cultured in insulin-/serum-deficient medium as indicated before the stimulation with 20 µM FSK for 2 h. Expression of *G6PC* was measured with qPCR. (*a*–*c*) Gene of interest was normalized to the housekeeper gene *TBP* and expression levels are shown as fold change over control (*n* = 3; mean ± s.d.; *p < 0.05, con versus FSK; ^#^*p* < 0.05, con versus con or FSK versus FSK).

The ability to generate glucose from gluconeogenic precursors is a key feature of hepatocytes and it has been shown that HepG2 cells express the gluconeogenic enzymes glucose-6-phosphatase (G6PC) and phosphoenolpyruvate carboxykinase (PCK1) and are able to produce glucose [20,21]. We tested if AML12 and THLE-2 cells also express *G6PC* and *PCK1* and respond to cAMP as an important driver of gluconeogenesis. Adenylyl cyclase activator forskolin was used to elevate intracellular cAMP concentrations. When HepG2 cells were starved of serum for 3 h and subsequently stimulated with forskolin for 2 h, a significant increase in *G6PC* and *PCK1* mRNA levels was measured (figure 6*a*,*b*). Serum starvation for 24 h before forskolin treatment resulted in a further increase in *G6PC* and *PCK1*, both in unstimulated and forskolin-stimulated conditions (figure 6*a*,*b*). In AML12 cells, forskolin treatment also induced *G6PC* mRNA, reaching higher levels after serum starvation for 24 h compared with 3 h (figure 6*c*). In contrast, in THLE-2 cells *G6PC* mRNA was at detection limit in unstimulated and stimulated conditions. Measured values of *PCK1* were at detection limit in both THLE-2 and AML12 cells. These results suggest that, in contrast to HepG2 cells and primary hepatocytes, these cells show an impaired/lost ability to express gluconeogenic enzymes.

### 2.4. Regulation of ANGPTL4 expression

**Figure 7.**
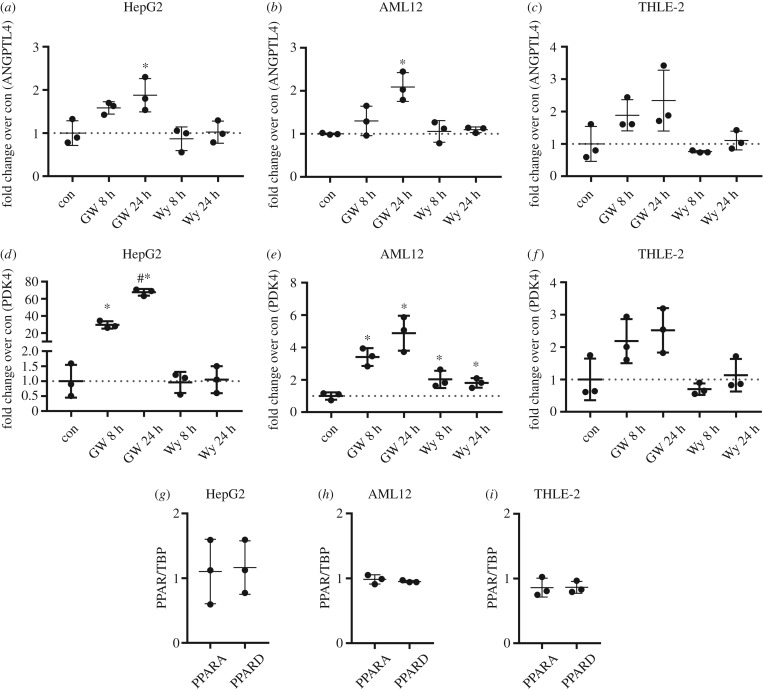
Expression levels of *ANGPTL4*, *PDK4*, *PPARA* and *PPARD* after stimulation with GW501516 or Wy-14643 in HepG2, AML12 and THLE-2 cells. Cells were cultured in their respective growth medium and stimulated with 1 µM GW501516 or 1 µM Wy-14643 for 8 or 24 h. Expression levels of *ANGPTL4*, *PDK4*, *PPARA* and *PPARD* were measured by qPCR. (*a*–*f*) Gene of interest was normalized for *TBP* and expression level is shown as fold changer over control (*n* = 3; mean ± s.d.; *p < 0.05, con versus 8 and 24 h GW501516 and Wy-14643; ^#^*p* < 0.05, 8 h versus 24 h GW501516). (*g*–*i*) Expression levels of *PPARA* and *PPARD* in the control sample was measured by qPCR and values are shown normalized for the housekeeper *TBP* (*n* = 3; mean ± s.d.).

Regulation of hepatokines angiopoietin-like protein 4 (ANGPTL4) and fibroblast growth factor 21 (FGF21) is driven by fatty acid-induced peroxisome proliferator-activated receptor (PPAR)α/δ activation and, as recently shown, also by cAMP-dependent pathways [22–24]. Since *FGF21* mRNA levels were at detection limit in AML12 and THLE-2 cells in all tested conditions, only expression of *ANGPTL4* was studied in the three cell lines. Stimulation with PPARδ agonist GW501516 for 8 and 24 h increased *ANGPTL4* mRNA levels in HepG2 and AML12 cells to a similar extent, with no significant effect in THLE-2 cells (figure 7*a*–*c*). Stimulation with PPARα agonist Wy-14643 did not alter *ANGPTL4* expression. Similar results were obtained for the PPARα/δ target gene *PDK4* (figure 7*d*–*f*), albeit PPARδ-dependent induction was more pronounced in HepG2 compared with AML12, while no significant increase was observable in THLE-2 cells. Stimulation with PPARα agonist showed a weak increase in *PDK4* only in AML12 cells. Since PPARα and PPARδ mRNA abundance was comparable in the three cell lines (figure 7*g*–*i*), it is unlikely that the non- or weak response to Wy-14643 in HepG2, AML12 and THLE-2 cells was due to low levels of PPARα.

**Figure 8.**
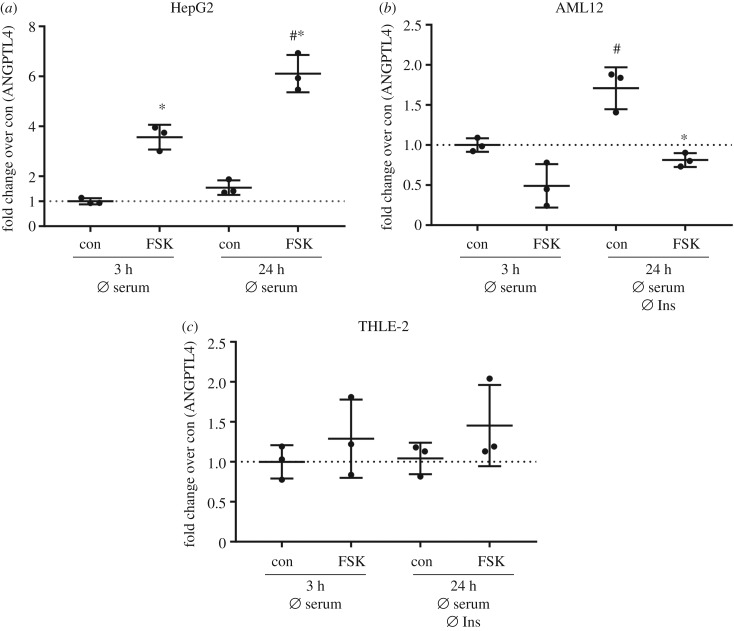
*ANGPTL4* expression in the investigated cell lines. (*a*–*c*) All cell lines were cultured with growth medium deficient for either insulin or serum or both as indicated before stimulation with 20 µM forskolin (FSK) for 2 h. *ANGPTL4* expression was measured by qPCR and normalized for housekeeper *TBP*. Expression level is shown as fold change over control (*n* = 3; mean ± s.d.; *p < 0.05, con versus FSK; ^#^*p* < 0.05, con versus con or FSK versus FSK).

Stimulation of HepG2 cells with forskolin resulted in a significant increase in *ANGPTL4* mRNA levels and the increase was even higher when cells were cultured for 24 h without serum (figure 8*a*). An upregulation of basal *ANGPTL4* mRNA levels after serum starvation for 24 h was observed in AML12 cells, but stimulation of these cells with forskolin led to a significant reduction of *ANGPTL4* (figure 8*b*)*.* No regulation in either condition was found in THLE-2 cells (figure 8*c*). Thus, only HepG2 cells resemble the *in vivo* regulation of *ANGPTL4* by cAMP [24].

## 3. Discussion

Human primary hepatocytes represent the ‘gold standard’ for studying hepatic insulin signalling and metabolic regulation on the cellular level [25]. Due to their limited availability, high costs when purchased, variation in quality due to variations in preparation and unstable phenotype, permanent cell lines such as HepG2 that are derived from a hepatocellular carcinoma are often used. Immortalized hepatic cell lines that originate from healthy liver are also available and might be of advantage compared with HepG2 cells since their metabolism should present a healthy state. Investigation of the insulin response of hepatic cell lines AML12 and THLE-2 that in revealed that substitution of the regular growth medium containing high insulin concentrations with an insulin-deficient medium for 24 h is necessary to achieve an insulin-induced AKT phosphorylation comparable to HepG2 cells. Thus, the high insulin concentrations in the regular culture medium of the manufacturer induced an insulin-resistant phenotype which is observable as reduced extent of AKT phosphorylation. Notably, withdrawal of insulin from the culture medium resulted in a time-dependent increase in the IR protein in AML12 cells. Cultivation in insulin-deficient medium for several passages might therefore induce further changes in the phenotype, probably resulting in a further increase in the insulin responsiveness of AML12 and THLE-2 cells, but this hypothesis needs to be tested with additional experiments. Other groups also reported insulin-induced AKT phosphorylation in AML12 cells when insulin was withdrawn from the culture medium [26,27], but for THLE-2 cells this is the first study reporting on insulin responsiveness. In primary hepatocytes, albeit cultured with 1 nM insulin for 16–18 h before stimulation, 0.1 nM insulin is sufficient to induce AKT phosphorylation [28], while 1–10 nM insulin is necessary in AML12 and THLE-2 cells. To conclude, in our experimental conditions, AML12 and THLE-2 cells are not superior over HepG2 cells with regard to AKT activation after acute insulin stimulation.

Analysis of IRS1, IRS2 and IR protein as key components of the insulin signalling cascade revealed differences in the protein amount in the three cell lines. The lower expression level of the IR protein in THLE-2 cells had no pronounced effect on insulin-induced AKT phosphorylation in our study but should be considered when insulin-dependent pathways are studied in this cell line. The band shape of IRS2 in THLE-2 cells looked broader than in samples from HepG2 or AML12 cells, where IRS2 bands were more concise. Differential posttranslational modifications of IRS2 such as phosphorylation, ubiquitinylation, acetylation and glycosylation in THLE-2 cells compared with HepG2 and AML12 cells might be an explanation for the different band shapes [26,27].

In diabetes research, the ability of hepatocytes to perform gluconeogenesis is of great interest since this pathway is dysregulated in insulin resistance and diabetes [29,30]. Gluconeogenic enzymes in HepG2 cells responded in our experimental set-up in a comparable manner to already published results [24]. However, AML12 and THLE-2 cells did not respond to forskolin stimulation to the same extent as did HepG2 cells. We found a similar regulation of *G6PC* in AML12 cells in response to forskolin but were not able to detect reliable levels of *PCK1* in AML12 cells. This is in some contrast to earlier data where *G6PC* and *PCK1* transcripts were detected in AML12 cells after stimulation with cAMP and dexamethasone [31] and a low but detectable glucose output was described [26]. However, the authors did not use labelled gluconeogenic precursors to validate whether the observed increase in extracellular glucose was derived from gluconeogenesis. To conclude, the overall ability of AML12 cells to perform gluconeogenesis might be limited and we were not able to detect any reliable *G6PC* or *PCK1* levels in THLE-2 cells.

The expression of hepatic ANGPTL4 was described to be regulated by PPARα, PPARδ and cAMP [24,32–34]. HepG2 and AML12 cells showed increased *ANGPTL4* mRNA levels after PPARδ activation, while there was a minor effect in THLE-2 cells. Also *PDK4* as another PPARδ target was increased in HepG2 and AML12 cells and no effect was observed in THLE-2 cells. Of note, the fold increase for *PDK4* expression was much higher in HepG2 cells compared with AML12 cells. In contrast to PPARδ activation, we observed no effects of PPARα agonist Wy-14643 on *ANGPTL4* expression in all investigated cell lines and only a weak effect on *PDK4* in AML12 cells. The non-response of HepG2 and THLE-2 is in contrast to Rakhshanderhoo *et al*. [32], who stimulated primary hepatocytes from mice and humans with Wy-14643 and measured a significant increase in *PDK4*. There might be two explanations: a higher concentration of Wy-14643 as we used might be necessary [32], and permanent cell lines might not react to Wy-14643 stimulation [35] as primary hepatocytes do. The mRNA levels of PPARα are similar between mouse and human [32]. In our investigated cell lines, PPARα levels were also similar and comparable to PPARδ. Therefore, weak expression of PPARα is not the explanation for the marginal effect of Wy-14643 on the cells.

The cAMP-dependent regulation of hepatic ANGPTL4 expression has been suggested based on *in vivo* data and was described in HepG2 cells [24] and in brown and white adipocytes [36]. We only observed higher *ANGPTL4* mRNA levels after forskolin stimulation in HepG2 cells, but not in AML12 or THLE-2 cells. Again, the two hepatocyte cell lines derived from healthy liver are not closer to the *in vivo* regulation as compared with HepG2 cells.

In summary, our data suggest that the regular culture conditions with high insulin concentrations generated an insulin-resistant phenotype in AML12 and THLE-2 cells. Although insulin withdrawal from the culture medium resulted in increased insulin responsiveness, AML12 and THLE-2 cells are not superior to HepG2 cells when insulin signalling is studied. Depending on the scientific question, it might be necessary to consider the different expression levels of the insulin receptor and its substrates. For studies on gluconeogenic gene and hepatokine expression, AML12 and THLE-2 cells appear not suitable since their expression profile did not resemble the *in vivo* situation.

## 4. Material and methods

### 4.1. Materials

GW501516 was obtained from Santa Cruz whereas Wy-14643 was purchased from Sigma Aldrich. All phosphatase inhibitor components were from Sigma, except sodium-ortho-vanadate, which was purchased from Applichem.

### 4.2. Cell culture

All cell lines were directly obtained from the manufacturer and were cultivated at 37°C with 5% CO_2_. HepG2 cells (ACC 180) were purchased from the DSMZ (Leibniz Institute DSMZ - German Collection of Microorganisms and Cell Cultures) and AML12 (CRL-2554™) and THLE-2 (CRL-2706™) cells were from ATCC. HepG2 cells were cultured in RPMI1640 (Lonza) supplemented with 10% FCS (Biochrom), 100 U ml^−1^ penicillin/streptomycin (Lonza) and 4 mM glutamine (Lonza). For propagation of cells 15 cm cell culture dishes (TPP) were used, and experiments were performed in 6-well plates (TPP). AML12 cells were grown in a 1 : 1 mixture of DMEM high glucose (Thermo Fischer)/Ham's F-12 with l-glutamine (Lonza) and supplemented with 10% FCS, 15 mM HEPES (Carl Roth), 40 ng ml^−1^ dexamethasone (Sigma Aldrich), 0.005 mg ml^−1^ (850 nM) human recombinant insulin (Sigma Aldrich), 5 ng ml^−1^ sodium selenite (Sigma Aldrich) and 0.005 mg ml^−1^ transferrin (Sigma Aldrich). AML12 cells were propagated in T-75 flasks (Greiner) and experiments were carried out in 6-well plates. For the cultivation of THLE-2 cells the BEGM Bullet Kit (CC-3170) from Lonza was used. The Bullet Kit contains BEBM Basal Medium and supplements. The final growth medium consists of the following: BEBM supplemented with 10% FCS, bovine pituitary gland extract, hydrocortisone, epidermal growth factor (EGF), insulin, triiodothyronine, transferrin, retinoic acid, 6 ng ml^−1^ human recombinant EGF (Sigma Aldrich) and 80 ng ml^−1^ o-phosphorylethanolamine (Sigma Aldrich). Measurement of insulin in the complete normal growth medium for THLE-2 cells revealed a concentration from 800–850 nM of insulin. THLE-2 cells require a special coating medium that consists of the following: RPMI1640 without glutamine supplemented with 0.01 mg ml^−1^ bovine serum albumin, (heat shock fraction, Sigma), 0.03 mg ml^−1^ type I collagen from bovine skin (Sigma) and 0.01 mg ml^−1^ fibronectin from human plasma (Sigma). THLE-2 cells were cultured in T-75 flasks and experiments were performed in 6-well plates; 4.5 ml of coating medium for a T-75 flask and 1 ml of coating medium for one 6-well plate were applied and culture dishes were incubated overnight in the incubator. Before seeding the coating medium was aspirated.

For experiments in insulin-deficient medium, AML12 and THLE-2 growth medium was prepared as described but no insulin was added to the medium. Therefore, minimal amounts of insulin in the insulin-deficient medium are derived from the insulin that is present in serum.

### 4.3. Stimulation

Serum was withdrawn from the culture medium of all three cell lines for 3 h before acute stimulation with 1, 10 or 100 nM insulin for 10 min. AML12 and THLE-2 cells were grown either in normal growth medium containing the recommended amount of insulin, or were cultured for 24 and 48 h without additional insulin. When forskolin was used to stimulate cells, they were cultured without serum for 3 or 24 h and in the case of AML12 and THLE-2 cells insulin was also withdrawn for 24 h before stimulation with 20 µM forskolin for 2 h. Stimulation with PPAR agonists was performed in cells grown in their respective growth medium with all recommended additives including insulin. PPAR agonists were used with a concentration of 1 µM and incubation times were 8 or 24 h. All independent experimental replicates were performed as technical duplicates.

### 4.4. Cell lysis, SDS-PAGE, western blot, protein detection

To harvest samples, cells were washed with cold PBS and lysed in RIPA buffer (25 mM Tris, 150 mM NaCl, 0.5% sodium deoxycholate, 0.1% SDS, 1% Triton-X-100, pH 7.6) supplemented with phosphatase inhibitors (100 mM sodium fluoride, 50 mM sodium pyrophosphate, 100 mM sodium ortho-vanadate and 100 mM β-glycerophosphate) and cOmplete™ Mini EDTA-free Protease Inhibitor (Roche). Samples were centrifuged for 5 min at 20 000*g* at 4°C and protein concentration was determined using a BCA Protein Assay Kit (Pierce) according to the manufacturer's instructions. 15–20 µg protein was separated on 5–15% reducing gradient gels (running buffer: 200 mM glycerol, 0.1% SDS, 25 mM Tris) and transferred to PVDF membranes with semi-dry western blot. After blocking unspecific binding sites, membranes were incubated overnight with primary antibodies: p-Thr-308, rabbit polyclonal, Cat. 9275, Cell Signaling; p-Ser-473, rabbit polyclonal, Cat. 9271, Cell Signaling; AKT, mouse monoclonal, Cat. 610861, BD Biosciences; GAPDH, mouse monoclonal, Cat. 8245, Abcam; IRS1, rabbit polyclonal, Cat. 06248, Millipore; IRS2, rabbit polyclonal, Cat. 3089, Cell Signaling; and insulin receptor protein chain β, rabbit monoclonal, Cat. 3025, Cell Signaling. Membranes were washed 3 × 15 min before incubation with secondary antibody for 2 h at room temperature. Secondary antibodies were all from LI-COR: IRDye^®^ 680LT donkey anti-rabbit IgG (H+L), Cat. 926-68023; IRDye^®^ 800CW donkey anti-mouse IgG (H+L), Cat. 926-32212; IRDye^®^ 800CW goat anti-rabbit IgG (H+L), Cat. 926-32211; and IRDye^®^ 680RD goat anti-mouse IgG (H+L), Cat. 925-68070. Membranes were scanned with the Odyssey Western Blot Scanner from LI-COR and analysed with Image Studio Lite (LI-COR).

### 4.5. RNA extraction, cDNA synthesis and quantitative real-time PCR

RNA was isolated by using the RNeasy Kit (Qiagen) according to the manufacturer's instructions. cDNA was transcribed from 0.5–1 µg RNA using Transcriptor First Strand cDNA Synthesis Kit (Roche). qPCR was performed in a 96-well format in a LightCycler 480 (Roche) with QuantiFast SYBR Green PCR Mix (Qiagen). By purifying PCR product with MiniElute PCR Purification Kit (Qiagen), standards for each primer were generated. Standard concentrations ranged from 5 ag µl^−1^ to 0.5 ng µl^−1^ and qPCR data were analysed using the LightCycler480 software. All QuantiTect Primer Assays were from Qiagen: human *ANGPTL4* (Cat. QT00003661), human *G6PC* (Cat. QT00031913), human *PCK1* (Cat. QT00001197), human *TBP* (Cat. QT00000721), human *PDK4* (Cat. QT00003325), human *PPARA* (Cat. QT00017451), human *PPARD* (Cat. QT00078064), mouse *Angptl4* (Cat. QT00139748), mouse *G6pc* (Cat. QT00114625), mouse *Pck1* (Cat. QT00153013), mouse *Tbp* (Cat. QT00198443), mouse *Pdk4* (Cat. QT00157248), mouse *Ppara* (Cat. QT00137984) and mouse *Ppard* (Cat. QT00166292). In this paper only the human abbreviation for the respective gene is used.

### 4.6. Statistical analysis

Two-tailed unpaired student's *t*-test was used to compare two groups and *p* < 0.05 was considered statistically significant.

## Data accessibility

All relevant data are included in the paper.
